# Assessment of cardiac RF ablation lesions by DCE-MRI

**DOI:** 10.1186/1532-429X-16-S1-P155

**Published:** 2014-01-16

**Authors:** Sathya Vijayakumar, Ravi Ranjan, Kyungpyo Hong, Daniel Kim, Nassir F Marrouche, Eugene G Kholmovski

**Affiliations:** 1 Surgical Services Division, Intermountain Healthcare, Salt Lake City, Utah, USA; 2 CARMA Center, University of Utah, Salt Lake City, Utah, USA; 3 UCAIR, Dept. of Radiology, University of Utah, Salt Lake City, Utah, USA

## Background

Radiofrequency (RF) ablation of myocardial tissue is a clinically acceptable therapy for atrial fibrillation and ventricular tachycardia. LGE-MRI has been used to assess immediately post-ablation RF lesions. It was shown that appearance of acute lesions in LGE-MRI changes with time after contrast injection [1,2]. Dickfeld, et al. [1] studied ventricular RF lesions delivered epicardially. They have shown that lesion core corresponds to regions of no-reflow with enhancement only at the boundaries in early LGE. Enhancement was shown to propagate inside lesion core in late LGE scans. Whereas, presence of considerable enhancement in regions of edema surrounding the no-reflow core of acute atrial lesions was shown in [2]. This obvious difference between these works was investigated and correspondence between acute no-reflow and 3 months post ablation scar was studied.

## Methods

RF ablations were performed in 5 mongrel dogs using ThermoCool, SmartTouch catheter (Biosense Webster, Diamond Bar, CA) according to protocols approved by the local IACUC. All MRI studies were performed on 3T Verio scanner (Siemens Healthcare, Erlangen, Germany). Endocardial RF ablations in four cardiac chambers were performed in the electrophysiology suite to create distinct lesions. The animals were then moved to the MRI suite. The study began with T2-weighted MRI, followed by contrast injection (0.15 mmol/kg,, MultiHance (Bracco Diagnostic Inc., Princeton, NJ)). 3D dynamic contrast enhanced (DCE) imaging of the whole heart was performed with following imaging parameters: respiratory navigated, ECG gated, saturation recovery prepared GRE sequence with spatial resolution = 1.25 × 1.25 × 2.5 mm, TR/TE = 2.9/1.4 ms, flip angle = 10o, temporal resolution of 2-3 minutes depending on respiration and heart rate, 8-10 frames. The MRI study was repeated 3 month post-ablation to visualize permanent lesions.

## Results

**Figure 1:**
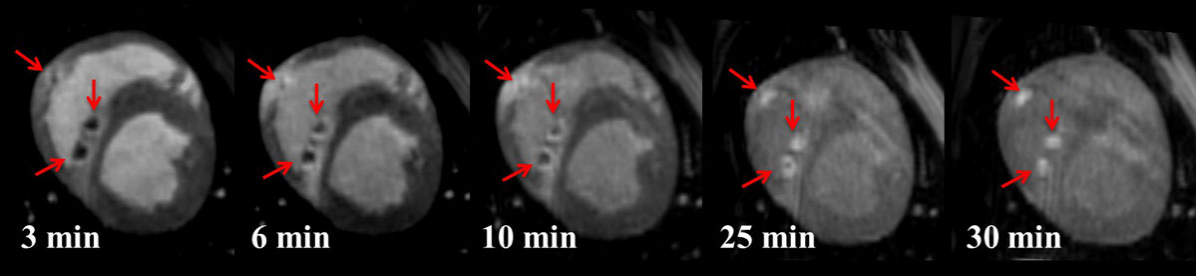
**Example of DCE-MRI of acute ventricular lesions**. Red arrows indicate the lesions.

**Figure 2:**
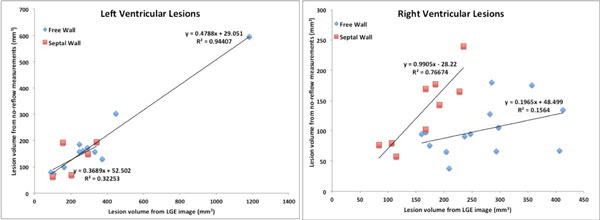
**Correlation between volume of no-reflow from acute studies with volume of permanent scar from 3-month post-ablation study**.

Figure 1 shows a series of DCE-MRI images of 3 representative acute ventricular lesions. Three distinct regions can be detected in the images. Significant enhancement is clearly observed in regions of edema surrounding lesion core (no-reflow regions in 1st frame) in the first 3 frames. Correlation between volume of no-reflow from acute study and volume of permanent scar from 3-month post-ablation study is shown in Figure 2.

## Conclusions

Our study shows that DCE-MRI may be used to differentiate between 3 distinct tissue types in acute post RF ablation studies: lesion core, edema, and normal myocardium. Our enhancement pattern agrees with previously demonstrated results for atrial lesions [2]. A possible explanation for difference in observation seen in [1] and our results could be the endocardial ablation as opposed to the epicardial ablation performed in [1]. No-reflow from acute studies overestimates the final lesion size at 3 months post ablation. These results may be explained by myocardial swelling due to edema in acute studies which resolve in the scar tissue observed in 3-month post-ablation scans.

## Funding

This study was funded in part by BioSense Webster.
